# Development and validation of a nomogram integrating endometrial ultrasound parameters to predict clinical pregnancy in frozen-thawed embryo transfer cycles

**DOI:** 10.3389/fendo.2026.1775977

**Published:** 2026-04-17

**Authors:** Qianyi Wang, Xu Yan, Jiale Ma, Zhifeng Sun, Honglu Diao, Pei Hu, Xiaoyi Wang, Ying Zhang, Yueyue Hu

**Affiliations:** 1 Reproductive Medicine Center, Renmin Hospital, Hubei University of Medicine, Shiyan, China; 2 Hubei Clinical Research Center for Reproductive Medicine, Renmin Hospital, Hubei University of Medicine, Shiyan, China; 3 The Third Medical College, Hubei University of Medicine, Shiyan, China; 4 Hubei Key Laboratory of Embryonic Stem Cell Research, Hubei University of Medicine, Shiyan, China; 5 Biomedical Engineering College, Hubei University of Medicine, Shiyan, China; 6 Department of Ultrasound Imaging, Renmin Hospital, Hubei University of Medicine, Shiyan, China

**Keywords:** frozen--thawed embryo transfer, nomogram, pregnancy outcomes, ultrasound, uterine peristalsis

## Abstract

**Objective:**

To develop and validate a multivariable logistic regression prediction model integrating ultrasound-based determinants influencing clinical pregnancy outcomes following frozen-thawed embryo transfer (FET) and to establish a clinically applicable prediction model integrating morphological and functional endometrial parameters.

**Methods:**

This study conducted uterine endometrium ultrasound evaluations on 325 infertile patients before transplantation. According to the inclusion and exclusion criteria, 107 infertile women were excluded, and 218 patients were included in the subsequent analysis. To ensure robust model evaluation and adhere to TRIPOD guidelines, the 218 participants were randomly split into a training cohort (n=154, ~70%) for model development and an validation cohort (n=64, ~30%) for performance assessment. Baseline characteristics, hormone levels, and ultrasound parameters were compared between the two groups. Univariate analysis, collinearity diagnosis, and multivariate logistic regression was utilized to determine independent predictors and construct a nomogram. A nomogram model was constructed in the training cohort and validated in the independent validation cohort, supplemented by1,000 bootstrap iterations. The performance of the model was assessed through discrimination, calibration, decision curve analysis (DCA), and clinical impact curves (CIC).

**Results:**

Multivariate analysis identified endometrial thickness (ET; OR = 1.315, *P* = 0.037) on the day before transfer, resistance index (RI; OR = 0.014, *P* = 0.027), systolic velocity/diastolic velocity (S/D; OR = 0.531, *P* = 0.047), uterine peristalsis (UP) direction (OR = 0.598, *P* = 0.022), and UP frequency (OR = 0.653, *P* = 0.039) as independent predictors of pregnancy outcomes. The nomogram demonstrated good discrimination (AUC = 0.828) and satisfactory calibration (Hosmer–Lemeshow *P* = 0.91). DCA shows that the model has a net clinical benefit in the probability range of 10% to 80%, and the CIC plot confirmed excellent concordance between predicted and observed pregnancy probability.

**Conclusion:**

The developed nomogram exhibits strong performance and clinical utility in predicting the possibility of pregnancy following FET, providing a non-invasive and practical tool for individualized clinical decision-making.

## 1 Introduction

Infertility affects approximately 15%-20% of reproductive-age couples attempting to conceive worldwide and has increasingly emerged as a significant public health concern (1). Despite significant progress in assisted reproductive technology (ART), implantation failure continues to be an essential constraint for achieving successful clinical pregnancy following embryo transfer (2–4). Successful implantation is influenced by embryo quality, endometrial condition, and the coordinated interaction between their developmental processes (5). Although techniques such as the endometrial receptivity array (ERA), molecular biomarker analysis, and omics analyses can provide detailed evaluation (6). However, their invasiveness, high cost, and technical complexity limit their routine clinical applications. Therefore, finding non-invasive, clinically accessible, and cost-effective predictors is crucial for predicting clinical pregnancy outcomes.

Transvaginal ultrasonography offers a convenient and reproducible method to assess both the physiological and pathological states of the uterus and endometrium (7, 8). Some ultrasonic parameters, including ET, Doppler flow indices (PI, RI, S/D), and uterine peristalsis (UP) are considered to be related to implantation success (9–11). However, the results of these studies remain controversial, and as most studies only focus on a certain indicator alone and have evaluated these parameters in isolation. Consequently, the synergistic predictive value of combining multiple ultrasound indicators warrants further investigation. Consequently, at joint predictive performance of these ultrasonographic indicators remains unclear.

Given the complex, multifactorial nature of embryo implantation (12), relying on a single ultrasound indicator alone may not be sufficient to accurately predict. pregnancy outcomes. While several recent studies have attempted to construct comprehensive prediction models for clinical pregnancy, they exhibit specific limitations regarding parameter integration. For instance, Guo et al. developed a robust model based on a large retrospective cohort of over 11,000 IVF/ICSI cases, yet their predictors were primarily limited to baseline demographic and laboratory characteristics (13). Similarly, Zhu et al. established a nomogram specifically for women with poor ovarian response, focusing on ovarian reserve markers rather than endometrial functional status (14). Furthermore, Liu et al. proposed a predictive model tailored to single Day-6 blastocyst transfers, which valuable does not fully account for the dynamic mechanical and hemodynamic changes of the endometrium immediately prior to transfer (15). Most existing models rely heavily on static baseline variables or specific patient subgroups, often overlooking the real-time synergistic value of morphological and functional endometrial parameters.

In order to overcome these limitations, we evaluated the synergistic predictive value of multiple ultrasound indicators and constructed a nomogram to visualize their combined association with pregnancy outcomes. This study aims to provide a practical, non-invasive tool to help clinicians in evaluating the state of the endometrium and optimizing decision-making in FET cycles.

## 2 Materials and methods

### 2.1 Study selection and sample partitioning

This prospective study recruited infertile women undergoing FET cycles in the Reproductive Medicine Center of the People’s Hospital Affiliated to Hubei University of Medicine (Hubei, China) from February to December 2024. All participants received a standardized endometrial preparation regimen and received endometrial ultrasound evaluation one day prior to embryo transfer. A study ethics approval was obtained (Approval No. SYRMYY-2025-108), and informed consent was obtained from every subject.

**Figure 1:**
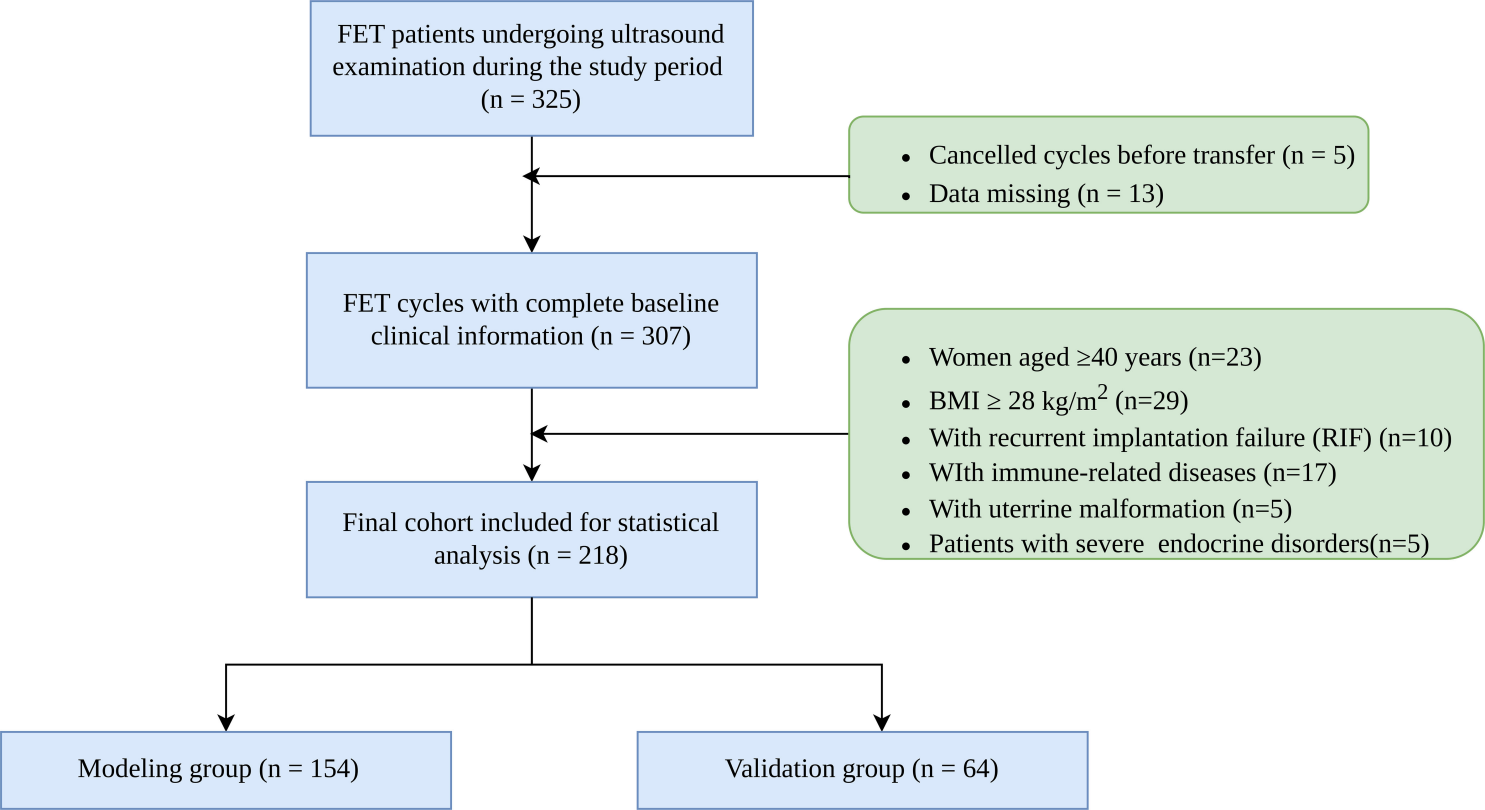
The flow diagram of study selection.

During the study period, a total of 325 patients undergoing FET who received ultrasound assessment were screened. Following the inclusion and exclusion criteria, 218 patients were ultimately included in the final analysis. Adhering to standard prediction model research guidelines, the total sample (N = 218) was randomly partitioned into a training cohort (n=154, 70.2%) used for variable selection and model construction, and an validation cohort (n=64, 29.8%) used to evaluate the model’s generalizability and predictive accuracy (**Figure 1**). Based on the results of the pregnancy outcome, the training cohort was divided into the pregnancy cohort and the non-pregnancy cohort. Clinical pregnancy outcomes were confirmed through telephone follow-ups to guarantee data accuracy and completeness.

### 2.2 Endometrial preparation and embryo transfer

All subjects enrolled in this study underwent endometrial preparation using a gonadotropin-releasing hormone agonist (GnRH-a) pituitary downregulation regimen combined with hormone replacement therapy (HRT). The specific protocol is as follows: On day 3 of the menstrual cycle, an intramuscular injection of a long-acting GnRH-a (Triptorelin, Ferring, Germany) was administered. After downregulation, endometrial priming commenced with oral estradiol valerate (Progynova; Bayer, Germany), starting at 4 mg per day and gradually adjusted to 6–8 mg per day depending on endometrial thickness and hormone levels. Upon confirming an endometrial thickness of ≥7 mm (minimum 16 days of estrogenization), secretory transformation was induced using 90 milligrams of vaginal progesterone sustained-release gel (Crinone; Merck Serono, Germany) combined with 20 milligrams of oral dydrogesterone (Duphaston; Sanofi, Netherlands).

Transvaginal ultrasonography was performed one day before embryo transfer to assess endometrial status. FET was performed 3 to 7 days after the initiation of progesterone transformation: cleavage-stage embryos were transferred on 3rd day, and blastocysts on 5th day. For patients with a displaced window of implantation (WOI) identified by ERA, embryo transfer timing was adjusted to day 6 or 7.

### 2.3 Observational indicators

#### 2.3.1 Baseline characteristics

maternal age, body mass index (BMI), antral follicle count(AFC), category of infertility (primary or secondary), pregnancy history, delivery history, abortion history, menstrual cycle length, and age at menarche, as well as the number and developmental stage of transferred embryos.

#### 2.3.2 Laboratory data

Fasting serum samples were collected via venipuncture at baseline (cycle days 2-3) between 7:00 AM and 9:00 AM. Hormone levels including anti-Müllerian hormone (AMH), follicle-stimulating hormone (FSH), luteinizing hormone (LH), estradiol (E_2_), and progesterone (P_4_) were measured using electrochemiluminescence immunoassay (ECLIA) on the Roche Cobas e601 analyzer (Roche Diagnostics, Mannheim, Germany). The inter-assay coefficients of variation (CV) were <5% for all hormones, with a specific CV of 4.2% for P_4_. Daily quality control was performed using commercial control materials (Bio-Rad Laboratories, Hercules, CA, USA). To evaluate the uterine environment during the preparation cycle, E_2_ and P_4_ levels were re-assessed in serum on two key timepoints: the day of progesterone initiation (endometrial transformation) and one day prior to FET. Crucially, the pre-FET hormonal measurement were synchronized with transvaginal ultrasound assessments to ensure temporal consistency. All analyses were conducted in the central laboratory of Renmin Hospital, Hubei University of Medicine, which participates in national external quality assessment programs.

#### 2.3.3 Ultrasound measurements

A comprehensive ultrasound examination was performed one day before embryo transfer. Parameters included endometrial thickness (ET) and volume (EV); endometrial blood flow indices including peak systolic velocity (PSV), end-diastolic velocity (EDV), pulsatility index (PI), resistance index (RI), and systolic/diastolic ratio (S/D); bilateral uterine artery PI, RI, S/D; and uterine peristalsis waves (frequency and direction).

#### 2.3.4 Clinical pregnancy

Defined by a positive serum β-hCG test 14 days after embryo transfer. If hCG levels remain elevated and menstruation does not occur, a transvaginal ultrasound is performed between 30 and 37 days following the transfer. The diagnosis of clinical pregnancy can be confirmed when the ultrasound image shows a gestational sac within the uterine cavity, containing an embryonic pole with detectable cardiac activity.

To eliminate inter-observer variability, all transvaginal ultrasound examinations were conducted by the same senior sonographer with over 10 years of experience in reproductive ultrasonography, who was blinded to the patients’ clinical information. Each parameter was measured in triplicate, and the average was calculated. The intra-observer CV was maintained at <5%, ensuring high data reproducibility. Uterine contraction activity was assessed according to the classification described by Ijland (16).

### 2.4 Statistical analysis

All data were randomly divided into a training set and a validation set at a ratio of 7:3. Normality of quantitative variables was evaluated using the Kolmogorov-Smirnov test. Normally distributed data were expressed as mean ± standard deviation (Mean ± SD), while non-normally distributed data were given as median with interquartile range (IQR) [M (Q1, Q3)]. Categorical data were reported as frequencies and percentages [n (%)]. Differences between groups were compared using the Student’s t-test or Mann-Whitney U test for continuous variables, and the Chi-square (χ²) test or Fisher’s exact test for categorical variables. First, a univariate logistic regression was performed to assess the statistical significance of each factor influencing the pregnancy outcomes. To address multicollinearity among the variables, the variance inflation factor (VIF) was employed for variable selection. Subsequently, variables exhibiting no multicollinearity (VIF<5) were entered into multivariate logistic regression analysis to identify independent predictors of pregnancy outcomes. Results were reported as odds ratio (OR) with 95% confidence interval (CI). A pregnancy prediction model was developed based on the above results, and the results were visualized using a nomogram. The model’s discriminative ability was quantified using the AUC. Calibration graphs were generated to assess the consistency between predicted probabilities and actual results. The model’s application value in a clinical setting was examined using DCA and CIC. Analyses were performed using SPSS 23.0 and R software version 4.4.3. Significance was set at *P* < 0.05 (two-tailed).

## 3 Results

### 3.1 Comparison of baseline characteristics between groups

A total of 218 patients were ultimately included in this study. The dataset was randomly divided into a training set (n=154) and a validation set (n=64) using a ratio of 7:3. Baseline characteristics were comparable between the training and validation cohorts (all P > 0.05), indicating successful randomization (**Table 1**).

**Table 1:** Clinical characteristics analysis of the modeling and validation set.

Variables	Modeling group (n=154)	Validation group (n=64)	*t*/χ^2^	*P*
Age, year	34.00 (30.00,36.00)	32.00 (30.00,35.00)	-1.613	0.109
BMI, kg/m^2^	22.59 (20.83,24.56)	22.10 (20.63,23.73)	-1.539	0.126
Antral follicle count	11.00 (6.00,17.00)	10.00 (8.00,16.00)	-0.307	0.759
AMH, ng/mL	3.57 (1.93,3.57)	3.40 (1.88,6.52)	-0.022	0.983
Basal E2, pg/L	34.50 (25.75,45.00)	37.00 (28.00,54.75)	-0.600	0.549
Basal P, pg/L	0.54 (0.31,0.79)	0.42 (0.22,0.71)	-1.397	0.164
Basal FSH, IU/L	6.57 (5.68,8.71)	6.85 (5.59,9.43)	-0.130	0.897
Basal LH, IU/L	3.86 (3.00,5.56)	4.06 (3.14,5.93)	-0.196	0.845
E2 on the transformationday, pg/ml	194.50 (138.75,389.50)	173.50 (119.50,297.25)	0.264	0.792
P on the transformation day, pg/ml	0.17 (0.05,0.32)	0.12 (0.02,0.30)	-0.625	0.532
E2 on the day before embryo transfer, pg/ml	241.00 (165.25,448.50)	245.50 (153.25,561.00)	0.900	0.370
P on the day before embryo transfer, pg/ml	42.18 (5.01,80.90)	41.88 (5.93,82.56)	-0.679	0.498
Endometrial thickness onthe day of endometrial transformation, mm	10.00 (8.80,11.53)	10.30 (8.40,11.75)	0.572	0.568
Duration of infertility, year	3.50 (2.00,5.25)	3.00 (2.00,5.00)	-1.204	0.231
Gravidity history (G)			0.511	0.475
NO	32.5 (50/154)	37.5 (24/64)		
YES	67.5 (104/154)	62.5 (40/64)		
Parturition history (P)			0.089	0.765
NO	70.8 (109/154)	68.8 (44/64)		
YES	29.2 (45/154)	31.2 (20/64)		
Type of infertility, %			3.315	0.069
Primary infertility	47.4 (73/154)	60.9 (39/64)		
Secondary infertility	52.6 (81/154)	39.1 (25/64)		
Age at menarche,year	14.00 (13.00,14.00)	14.00 (13.00,14.00)	-0.347	0.730
Menstrual cycle duration,day	28.00 (28.00,36,00)	30.00 (26.50,31.00)	-1.563	0.120
Transferred embryo, %			0.035	0.851
1	64.3 (99/154)	65.6 (42/64)		
2	35.7 (55/154)	34.4 (22/64)		
Transferred embryo stage, %			2.094	0.148
blastocyst	13.0 (20/154)	6.3 (4/64)		
cleavage	87.0 (134/154	93.7 (60/64)		

BMI, Body mass index; AMH, AntiMullerian hormone; E2, estradiol; P, progesterone; FSH, follicular stimulating hormone; LH, luteinizing hormone.

### 3.2 Comparison the baseline characteristics of modeling cohorts

In the training cohort, patients were stratified into a pregnancy group (n=86) and a non-pregnancy group(n=68) according to the clinical pregnancy outcome. As shown in **Table 2**, no significant differences were observed between the two groups in most baseline clinical characteristics, with the exception of AFC and basal FSH, which exhibited significant intergroup variation(P<0.05). For other variables, including age, BMI, menstrual history (age and duration), infertility-related factors (type of infertility, gravidity, parity, and history of miscarriage), AMH, baseline hormone levels (E_2_, P_4_, and LH), serum hormone levels on the day of endometrial transformation and the day prior to embryo transfer, as well as embryo transfer characteristics (number of embryos transferred and developmental stage), no statistically significant differences were observed between the two groups (all *P* > 0.05), indicating good comparability. The homogeneity of these baseline characteristics effectively minimizes the potential risk of confounding bias within the study.

**Table 2:** Clinical characteristics analysis of the pregnancy and non-pregnancy groups.

Variables	Non-pregnancy group (n=68)	Pregnancy group (n=86)	*t*/χ^2^	*P*
Age, year	35.00 (31.00,37.00)	33.00 (30.00,36.00)	0.896	0.372
BMI, kg/m^2^	22.04 (20.59,24.17)	22.97 (20.94,24.97)	-1.012	0.313
Antral follicle count	9.00 (6.00,14.00)	13.00 (8.00,18.00)	-1.982	0.049*
AMH, ng/mL	3.33 (1.88,4.74)	3.68 (2.27,5.81)	-1.425	0.156
Basal E2, pg/L	34.50 (25.00,46.25)	34.50 (26.00,45.00)	-1.405	0.163
Basal P, pg/L	0.58 (0.35,0.75)	0.51 (0.30,0.86)	-0.609	0.544
Basal FSH, IU/L	7.12 (5.82,9.65)	6.37 (5.55,7.72)	2.180	0.031*
Basal LH, IU/L	3.72 (2.89,5.62)	3.89 (3.08,5.56)	-0.500	0.618
E2 on the transformationday, pg/ml	195.50 (143.50,320.50)	180.50 (129.50,406.75)	-0.998	0.320
P on the transformation day, pg/ml	0.22 (0.08,0.36)	0.14 (0.03,0.29)	1.642	0.104
E2 on the day before embryo transfer, pg/ml	280.50 (155.00,512.50)	229.00 (170.75,377.94)	1.321	0.189
P on the day before embryo transfer, pg/ml	41.29 (3.95,88.57)	43.49 (5.68,76.39)	1.066	0.290
Endometrial thickness onthe day of endometrial transformation, mm	9.85 (8.80,11.05)	10.20 (8.80,11.65)	-0.587	0.558
Duration of infertility, year	3.00 (2.00,5.00)	4.00 (2.00,6.00)	-0.940	0.349
Gravidity history (G)			0.001	0.978
NO	32.4 (22/68)	32,6 (28/86)		
YES	67.6 (46/68)	67.4 (58/86)		
Parturition history (P)			0.0021	0.963
NO	70.6 (48/68)	70.9 (61/86)		
YES	29.4 (20/68)	29.1 (25/86)		
Type of infertility, %			1.893	0.169
Primary infertility	41.2 (28/68)	52.3 (45/86)		
Secondary infertility	58.8 (40/68)	47.7 (41/86)		
Age at menarche,year	14.00 (13.00,14.00)	14.00 (13.00,14.00)	1.643	0.103
Menstrual cycle duration,day	30.00 (28.00,30.75)	30.00 (28.00,37.75)	-0.803	0.423
Transferred embryo, %			0.190	0.663
1	66.2 (45/68)	62.8 (54/86)		
2	33.8 (23/68)	35.1 (32/86)		
Transferred embryo stage, %			3.050	0.054
blastocyst	19.1 (13/68)	8.1 (7/86)		
cleavage	80.9 (55/68)	91.9 (79/86)		

BMI, Body mass index; AMH, Anti Mullerian hormone; E2, estradiol; P, progesterone; FSH, follicular stimulating hormone; LH, luteinizing hormone. *p<0.05.

### 3.3 Comparison the pre-transfer ultrasound parameters of modeling cohorts

**Table 3** summarizes the ultrasound parameters measured one day before FET. There were no significant statistical differences between both groups as far as EV, PSV, EDV, and PI. Similarly, concerning the blood supply to the uterus, no significant disparities in the Doppler indices (PI, RI, S/D) of the bilateral uterine arteries between pregnancy and non-pregnancy groups (*P* > 0.05). However, significant disparities in endometrial thickness and endometrial hemodynamic resistance were observed. Patients who achieved pregnancy had a significantly thicker endometrium (*P* = 0.001) and reduced vascular resistance, as evidenced by lower endometrial RI and endometrial S/D ratios compared to non-pregnant women (RI: 0.50 *vs*. 0.56; S/D: 2.09 *vs*. 2.45; both *P* < 0.001). In terms of uterine contractility, the pregnancy group had a more “quieter” uterus with significantly lower peristaltic frequency (1.00 *vs*. 2.00; *P* = 0.002). Directional analysis of peristaltic waves showed that favorable patterns—specifically cervix-to-fundus (CF) activity and no activity—were dominant in pregnancy group. In contrast, the non-pregnancy group exhibited a significantly higher percentage of adverse wave patterns, such as fundus-to-cervix (FC), random(R), or opposing waves(OP).

**Table 3:** The patients’ endometrial ultrasonographic parameters one day before FET.

Variables	Non-pregnancy group (n=68)	Pregnancy group (n=86)	*t*/χ^2^	*P*
Endometrial thickness, mm	8.95 (8.00,10.20)	9.70 (8.98,11.13)	-3.508	0.001**
Endometrial volume, ml	3.10 (2.13,5.00)	3.65 (2.80,5.30)	-1.132	0.260
Endometrial PI	0.84 (0.63,0.99)	0.79 (0.63,0.96)	-0.020	0.984
Endometrial RI	0.56 (0.51,0.64)	0.50 (0.44,0.55)	4.434	0.000***
Endometrial PSV	7.99 (6.00,9.92)	7.76 (5.85,9.51)	1.060	0.291
Endometrial EDV	3.52 (2.78,4.38)	3.51 (2.78,4.28)	0.778	0.438
Emdometrial S/D	2.45 (2.06,3.14)	2.09 (1.88,2.35)	4.260	0.000***
Left uterine artery PI	2.31 (1.79,2.72)	2.29 (1.94,2.69)	-1.012	0.313
Left uterine artery RI	0.85 (0.81,0.88)	0.85 (0.81,0.88)	-0.597	0.552
Left uterine artery S/D	6.55 (5.11,8.06)	7.03 (5.35,8.25)	-0.258	0.797
Right uterine artery PI	2.21 (1.85,2.57)	2.27 (1.81,2.72)	-0.363	0.717
Right uterine artery RI	0.84 (0.81,0.87)	0.85 (0.81,0.88)	-1.067	0.288
Right uterine artery S/D	6.27 (5.05,7.91)	6.73 (5.25,8.47)	-0.783	0.435
Uterine peristalsis direction			15.706	0.003**
No activity (N)	11.8 (8/68)	12.8 (11/86)		
Cervix-to-fundus (CF)	33.8 (23/68)	62.8 (54/86)		
Fundus-to-cervix (FC)	41.2 (28/68)	19.8 (17/86)		
Opposing waves (OP)	5.9 (4/68)	2.3 (2/86)		
Random (R)	7.4 (5/68)	2.3 (2/86)		
Uterine peristalsis frequency, (times/min)	2.00 (1.00,3.00)	1.00 (1.00,2.00)	3.204	0.002**

PI, pulsatility index; RI, resistance index; PSV, peak systolic velocity; EDV, end-diastolic velocity; S/D, systolic/diastolic ratio. **p<0.01; ***p<0.001.

### 3.4 Multivariate logistic regression and collinearity diagnostics

Candidate predictors identified via univariate analysis in the training cohort (*P* < 0.05) included AFC, basal FSH, ET, RI, S/D, UP frequency, and UP direction(all *P* < 0.05). Multicollinearity tests among those candidate variables were conducted before including them in multivariate analysis. The results showed that tolerance values ranged from 0.730 to 0.929, and VIF between 1.077 and 1.370 (all < 5), indicating no significant multicollinearity among predictors (**Table 4**). Then these seven predictors were entered into multivariate logistic regression. As presented in **Table 5**, the impacts of AFC and basal FSH on pregnancy outcomes were no longer statistically significant (P > 0.05) and were therefore excluded from the final model. ET emerged as a significant protective factor that promotes pregnancy (OR = 1.315, 95% CI: 1.017-1.700, P = 0.043). In contrast, hemodynamic resistance (RI and S/D) and abnormal uterine peristalsis (frequency and adverse direction) were identified as independent risk factors of embryo implantation (all *P* < 0.05).

**Table 4:** Collinearity diagnostics of significant variables associated with pregnancy outcomes in modeling set.

Model	Unstandardized coefficients		Standaardizes coefficients	*t*	*Sig.*	Collinearity statistics	
	B	Std error	Beta			Tolerance	VIF
(Constant)	1.179	0.335		3.522	0.001		
AFC	0.003	0.005	0.046	0.612	0.542	0.853	1.172
Basal FSH	-0.025	0.011	-0.170	-2.266	0.025	0.860	1.162
RI	-0.718	0.300	-0.195	-2.391	0.018	0.730	1.370
S/D	-0.102	0.043	-0.186	-2.356	0.020	0.775	1.290
ET	0.044	0.022	0.152	2.040	0.043	0.876	1.141
UP frequncy	-0.083	0.036	-0.168	-2.310	0.022	0.918	1.090
UP direction	-0.086	0.039	-0.158	-2.189	0.030	0.929	1.077

RI, resistance index; S/D, systolic/diastolic ratio; ET, endometrial thickness; UP, uterine peristalsis; FSH, follicular stimulating hormone; AFC, antral follicle count.

**Table 5:** Multivariate logistic regression analysis of modeling set.

Variables	*B*	Std.Error	*Wald*	*P*	OR(95%*CI*)	
AFC	0.024	0.031	0.616	0.432	1.025 (0.964--1.088)
Basal FSH	-0.123	0.065	3.625	0.057	0.884 (0.779--1.004)
Endometrial thickness,mm	0.274	0.131	4.368	0.037	1.315 (1.017--1.700)
Endometrial RI	-4.271	1.936	4.864	0.027	0.014 (0.000--0.622)
Endometrial S/D	-0.632	0.318	3.951	0.047	0.531 (0.285--0.991)
Uterine peristalsis frequency, (times/min)	-0.427	0.207	4.273	0.039	0.653 (0.435--0.978)	
Uterine peristalsis direction	-0.514	0.224	5.268	0.022	0.598 (0.385--0.928)
Constant	3.539	1.905	3.451	0.063		

RI, resistance index; S/D, systolic/diastolic ratio; AFC, antral follicle count; FSH, follicular stimulating hormone.

### 3.5 Nomogram construction and validation

**Figure 2:**
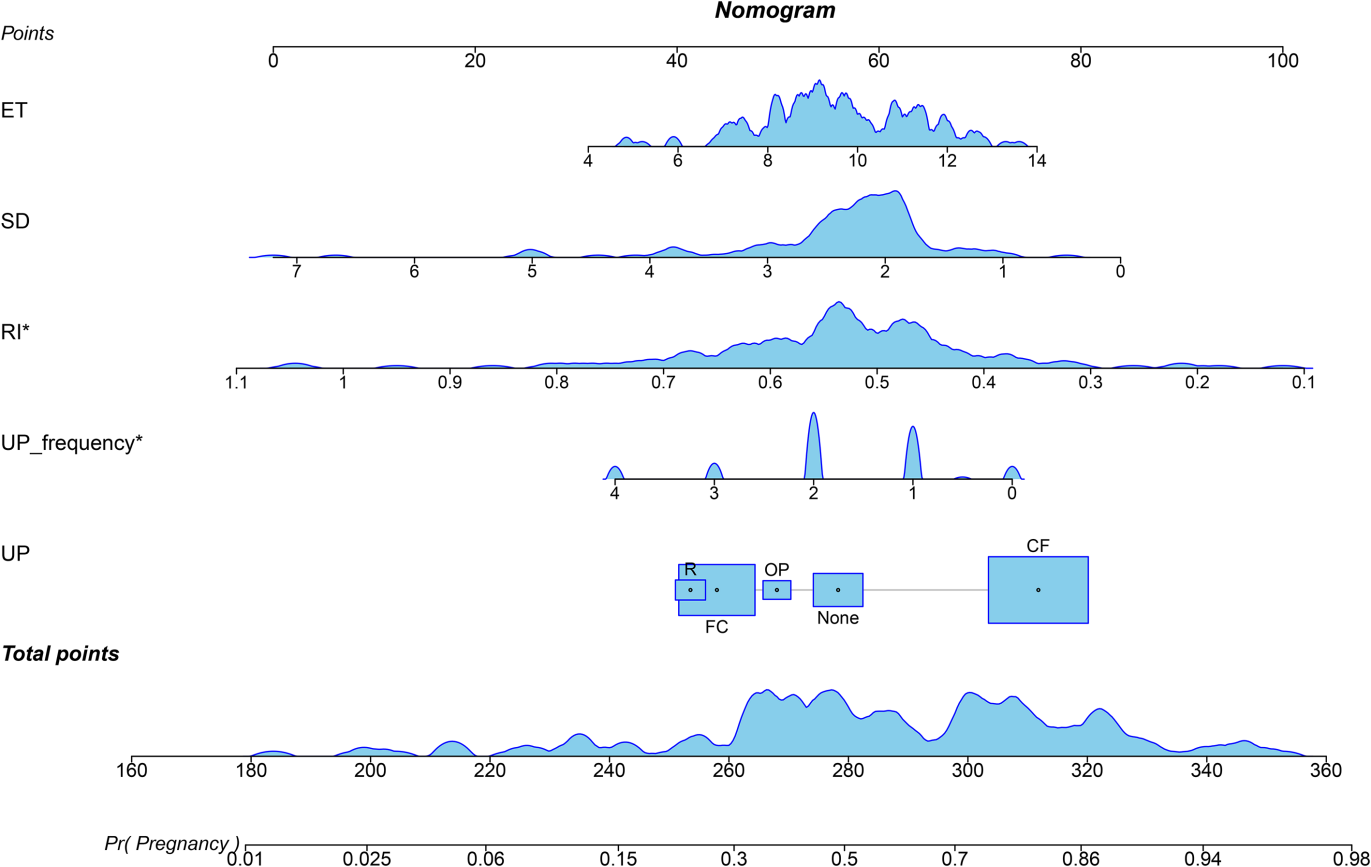
Nomogram model for predicting pregnancy probability in FET patients. The nomogram integrates five independent ultrasound predictors: endometrial thickness (ET), systolic/diastolic ratio (S/D), resistance index (RI), uterine peristalsis (UP) frequency, and UP direction. Each variable is assigned a corresponding “Points” scale by the model, ranging from 0 to 100. The sum of all points yields the “Total Points”. The probability vertically corresponding to the total score represents the likelihood of achieving clinical pregnancy for that individual.

**Figure 3:**
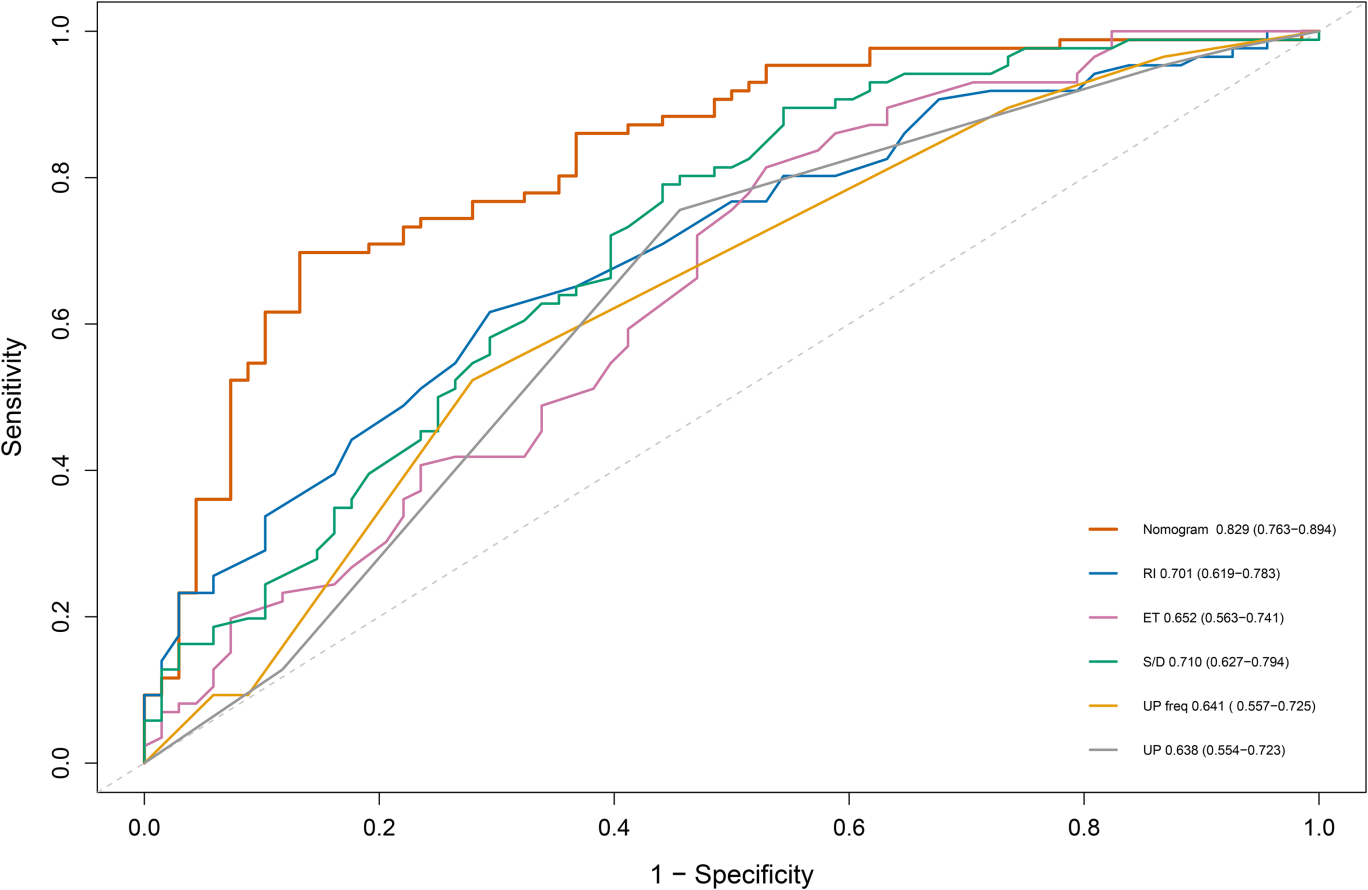
ROC curve of prediction models and various ultrasound indicators. The red curves hows the nomogram, which achieved the highest discriminative ability (AUC = 0.828, 95% CI 0.763-0.894). ROC curves for individual predictors are displayed for comparison. The gray diagonal line serves as a reference without discriminative value.

**Figure 4:**
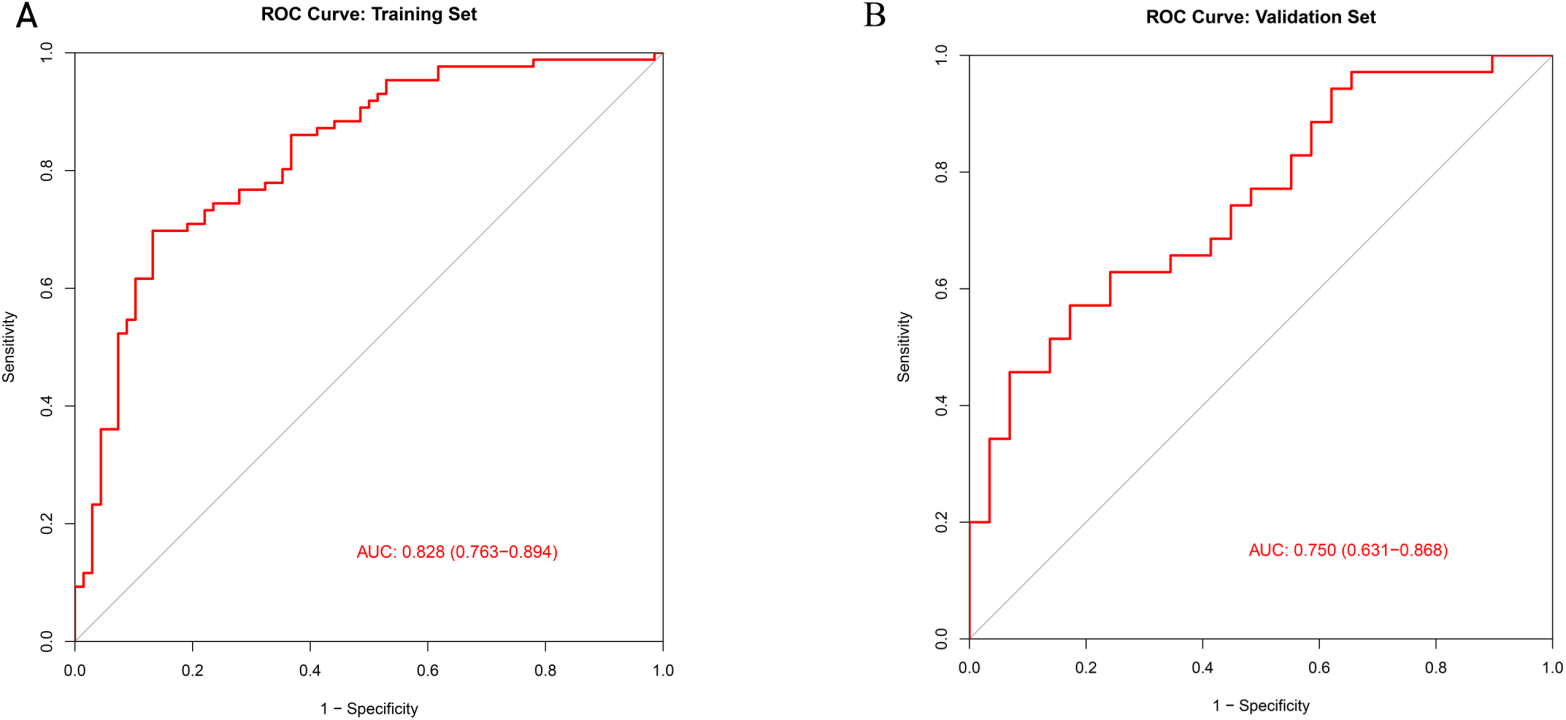
ROC curve for the training set and validation set. **(A)** shows the ROC curve of the training set and **(B)** shows the ROC curve of the test set. The X-axis represents specificity, and the Y-axis represents sensitivity. The AUC of the ROC curve for the modeling group was 0.828 (95% CI: 0.723-0.894). The AUC of the ROC curve for the validation group was 0.750 (95% CI: 0.631-0.868).

**Figure 5:**
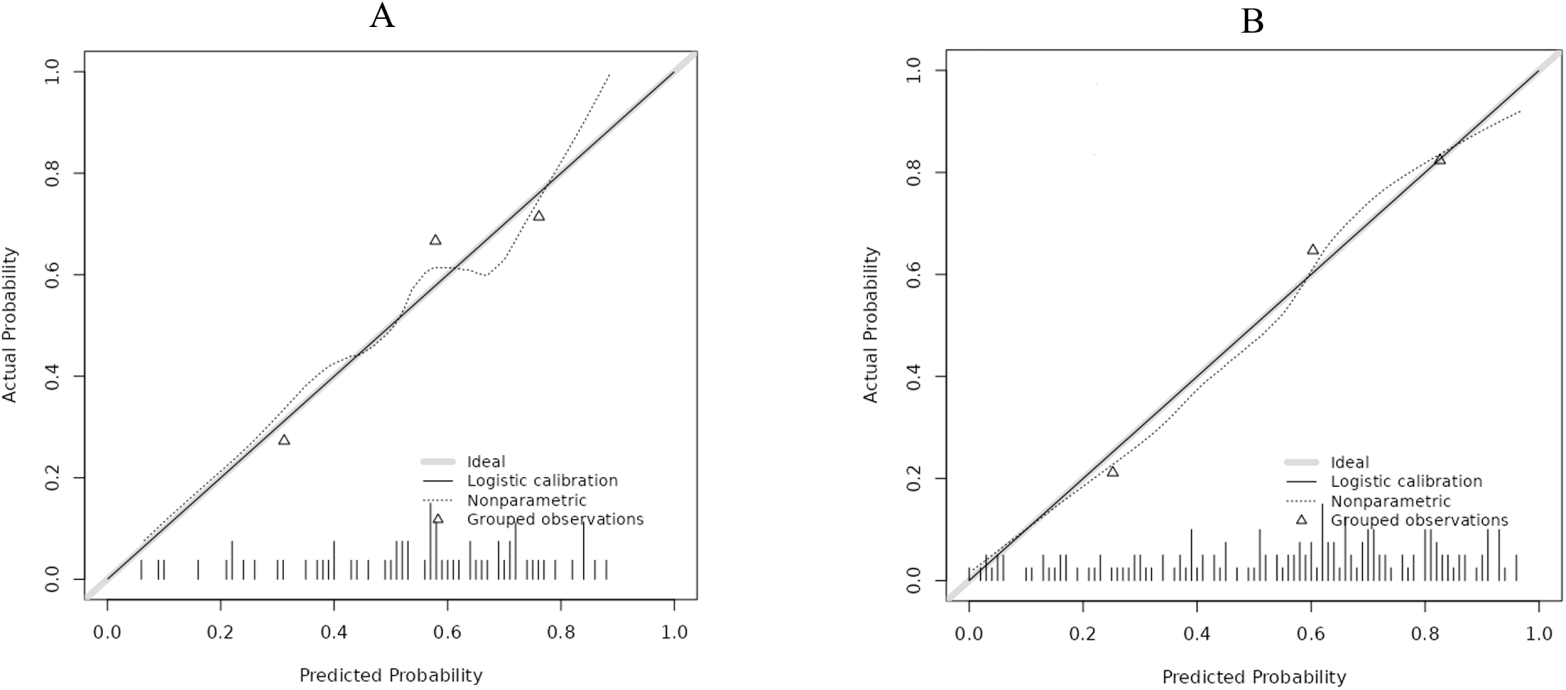
Calibration curve for the training set and validation set. **(A)** represents the training set, and **(B)** represents the test set. The X-axis represents the predicted probability of achieving clinical pregnancy, while the Y-axis shows the actual probability. The dashed line represents the actual values corresponding to the model’s predicted values, while the solid line denotes the bias-corrected estimate obtained through 1000 bootstrap samples. The gray diagonal line denotes the ideal reference line for perfect calibration. The excellent match of the bias-corrected curve with the ideal line signifies superior calibration performance of the nomogram.

**Figure 6:**
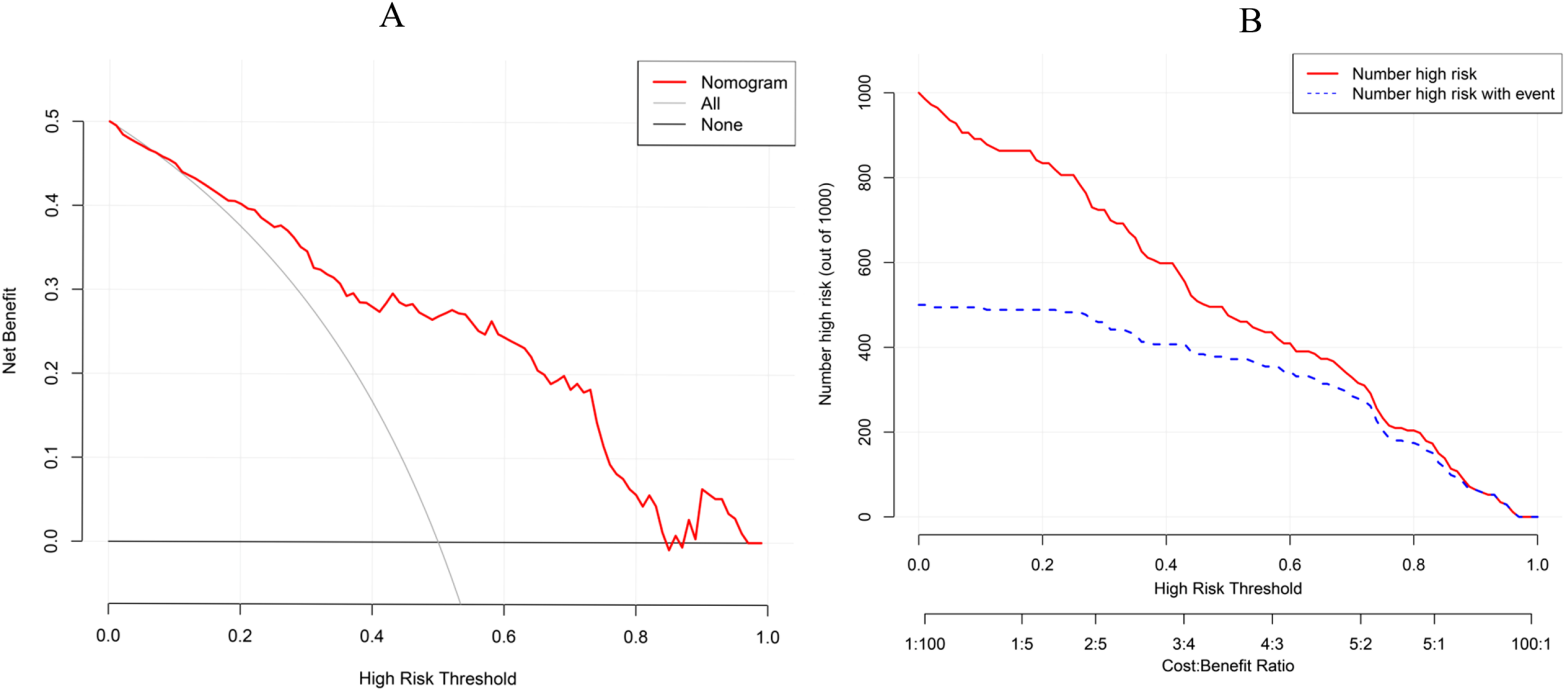
DCA and CIC of the prediction model. **(A)** represents model decision curve analysis. **(B)** represents the model’s clinical impact.

A nomogram was established incorporating 5 independent predictors: ET, RI, S/D, UP direction, and UP frequency (**Figure 2**). The model demonstrated excellent discrimination. In the training cohort, the AUC was 0.828 (95% CI: 0.763-0.894), outperforming the predictive value of individual ultrasound parameters (**Figure 3**). Crucially, in the validation cohort, the model maintained robust discriminatory power with an AUC of 0.750 (95% CI: 0.631-0.868) (**Figure 4**). The calibration curves revealed high consistency between the predicted probabilities and actual outcomes, characterized by the closeness between the ideal, bias-corrected, and apparent curves (**Figure 5**). This goodness-of-fit was further confirmed by the Hosmer-Lemeshow test (Training: χ² = 3.37, P = 0.91, Validation: χ² = 12.57, P = 0.73), where the non-significant P-value (> 0.05) suggested no significant deviation between predicted and observed risks. Moreover, DCA and CIC analyses were carried out to further examine the model’s clinical application value in forecasting pregnancy outcomes(**Figure 6**). DCA demonstrated that applying the nomogram to inform clinical decisions provides more net benefit than either the “treat-all” or “treat-none” scheme within a threshold probability range of 10%-80%. Furthermore, CIC confirmed the model’s high clinical validity, showing a favorable cost-benefit ratio across risk thresholds of 0.2-0.8. These validation indicators collectively indicate that the proposed nomogram has excellent predictive accuracy and substantial clinical utility for guiding FET strategy.

## 4 Discussion

In the present study, we constructed and validated a predictive nomogram for FET pregnancy outcomes by integrating multiple endometrial ultrasound parameters. Our multivariate analysis showed that five independent predictors were ET, RI, S/D, UP direction, and UP frequency. The findings indicated that a thicker endometrium, low-resistance blood flow, and favorable uterine peristalsis (specifically cervix-to-fundus waves or no contraction) were all associated with higher pregnancy rates. The nomogram exhibited strong discriminatory ability (AUC = 0.828) and good calibration (Hosmer–Lemeshow *P* = 0.91). DCA confirms its net benefit across a wide range of threshold probabilities (10%-80%). The results highlight the value of a comprehensive ultrasound assessment—including morphology, hemodynamics, and peristaltic activity—that can provide a reliable, non-invasive tool for clinical prognostication.

The predictive power of our model is biologically underpinned by the critical roles of ET, vascular resistance, and uterine contractility in embryo implantation. A thicker endometrium means more glandular development, increased vascularization, and abundant glycogen reserves, creating a nourishing microenvironment for embryonic attachment and growth (17). In terms of endometrial hemodynamics, lower RI and S/D values reflect reduced vascular resistance and better endometrial perfusion. This optimized hemodynamic environment facilitates the more readily delivery of oxygen and nutrients while faster removal of metabolic waste, promoting trophoblastic invasion (18, 19). Besides morphology and perfusion, uterine peristalsis is increasingly acknowledged as an important control over embryo transport and implantation (20). CF waves or no contraction of the uterus are favorable for rapid movement of the embryo to the ideal implantation site. Conversely, high frequency or reverse peristalsis might mechanically hinder implantation or induce embryo expulsion (20). Collectively, the present study indicates that a multidimensional assessment can provide greater predictive accuracy in comparison to a single parameter assessment.

Whereas ET is a common marker to evaluate outcomes of ART (21), its prognostic value is often contested (22). Our data showed a significant positive association, where a relatively thicker endometrium was an independent predictor of successful implantation. Namely, each 1 mm increase in ET translated to a 24.9% increase in conception likelihood. This observation is consistent with substantial evidence which suggests that adequate endometrial thickness is a prerequisite for pregnancy (23, 24), with a thicker endometrium usually translating into improved implantation rates, live birth rates, and lower miscarriage risks (25). Interestingly, there is clinical evidence which shows that pregnancy success rate is even lower than 30% if the ET is < 6 mm (26). The potential mechanism may lie in the functional layer becoming thinner or even absent, forcing the embryo to implant closer to the basal spiral arteries. The high blood perfusion and high oxygen tension environment in this region readily induce the generation of reactive oxygen species (ROS), which in turn exert inhibitory effects on embryo implantation and early development (27). However, the “thicker is better” notion has its own limitations. Evidence shows a nonlinear relationship, where the implantation and the pregnancy rate may paradoxically decline when the endometrium becomes excessively thick (>14 mm) (28). Moreover, a meta-analysis by Kasius et al. argued ET had low sensitivity and specificity when considered as an independent predictor (26), which is likely attributable to inter-individual variability and different statuses in the transfer cycle. Taken together, while ET is an important piece of the assessment, its predictive power is probably modulated by other factors, the comprehensive model proposed in this study is needed.

Sufficient endometrial perfusion is a fundamental prerequisite for successful implantation and early embryonic development. Our multivariate analysis confirmed that endometrial blood flow held a critical weight in the predictive model. Specifically, the pregnant group had much lower endometrial RI and S/D values, reflecting a low vascular-impedance state that is conducive to embryo apposition and invasion. While our results support the findings of Tong et al. (29), they differ from those of Kim et al. (30). The discrepancies between studies may arise from variations in sample size, study populations, stimulation protocols, or uncorrected confounding factors (e.g., medication use, uterine surgery history, and parity) that affect vascular dynamics (31). A key finding of our study is the dissociation between systemic and local blood flow. While endometrial blood flow indices were predictive, the Doppler parameters of the bilateral uterine arteries did not show any significant difference between the groups. This indicates that uterine artery analysis cannot reflect the local variations of the implanted area (32). Physiologically, this might be explained by the blood flow characteristics of the spiral arteries—the terminal branches supplying nutrients for the endometrium (18, 33). The resistance in the uterine artery is normally controlled by the local microvascular network before reaching the endometrial layer (34). Hence, a high uterine artery resistance does not necessarily indicate insufficient local perfusion, and vice versa. Thus, we believe that direct assessment of endometrial microcirculation yields a more sensitive and biologically informative marker of implantation potential than global uterine artery indices (35).

Uterine peristalsis has recently emerged as a vital functional index of reproductive potential. Abnormal uterine contractility not only impairs embryo migration and intrauterine positioning (36) but is also associated with the cause of repeated implantation failure (RIF) (37). We have found that the pregnancy group predominantly exhibited cervix-to-fundus (CF) waves or no contraction patterns. This supports the theory of a “retention mechanism”, maintaining the embryo adhesion at the optimal implantation site (18, 29–40). From a mechanistic perspective, abnormal uterine contractility may stem from overexpression of oxytocin receptors (OTR) or dysregulated prostaglandin signaling during the mid-luteal phase (41, 42). Such hyperactivity, particularly fundus-to-cervix (FC) waves, can physically disrupt the embryo-endometrial interaction, and serve as a biological biological “expulsion pump” that displaces the embryo into the cervix or fallopian tubes, increasing the risk of implantation failure or ectopic pregnancy (43). As for clinical prediction point of view, we found in our multivariate analysis that UP frequency was a significant negative predictor; specifically, every decrease of 1 times/min was correlated with 38.3% enhance in the likelihood of pregnancy. In line with the previous meta-analyses (36), we found that frequencies exceeding 3 times/min are detrimental, whereas a lower frequency (1–2 times/min) is conducive to implantation (38) (44). So we believe it is essential to assess uterine contractions dynamics (both direction and frequency) to gain us an overall view of successful implantation (45).

AFC and basal FSH levels are traditionally regarded as classical indicators for evaluating ovarian reserve (46).This study demonstrates that while both variables were significant associated with clinical pregnancy outcomes in univariate analyses, their independent predictive value was no longer statistically significant after adjustment for endometrial characteristics in the multivariable model (P > 0.05). This phenomenon may primarily be explained by the fact that AFC and FSH predominantly reflect the primordial developmental potential of oocytes—the quality of the ‘Seed’. However, in FET cycles, embryos undergo rigorous morphological assessment or even preimplantation genetic testing prior to transfer, leading to a high degree of homogenization in embryo quality. Such artificial pre-selection reduces the discriminatory capacity of ovarian reserve markers in predicting pregnancy outcomes (47). Furthermore, most FET cycles frequently utilize HRT or pituitary downregulation protocols to artificially modulate the endometrial environment, thereby uncoupling endometrial development from the endogenous regulation of the patient’s hypothalamic-pituitary-ovarian axis (48). Consequently, when more temporally relevant and direct indicators of endometrial receptivity—such as blood flow parameters and endometrial peristaltic activity—are incorporated into a multivariate model, the predictive weight of AFC and FSH is further attenuated.

Despite the promising results, several limitations of this study must be acknowledged. First, the single-center design and relatively modest sample size may introduce selection bias. Second, a critical practical challenge lies in the technical expertise required for detailed ultrasound assessments. In this study, all uterine peristalsis and hemodynamic parameters were measured by a single senior sonographer with over 10 years of specialized experience to ensure data consistency. While this approach minimized inter-observer variability, it raises concerns regarding generalizability. Maintaining such a high level of operator expertise and consistency in routine clinical practice—across both commercial fertility centers and university hospitals—can be difficult. Consequently, the subjective nature of these measurements may hinder the widespread adoption of this nomogram in settings without specialized training. Third, our analysis relied on static parameters obtained immediately before transfer, potentially overlooking dynamic cyclical changes in endometrial receptivity. Finally, while internal validation yielded robust results, large-scale multicenter external validation is essential to confirm the model’s generalizability. Future studies should focus on developing standardized training protocols or integrating AI-assisted diagnostic tools to reduce operator dependency, thereby enhancing the objectivity and real-world applicability of these measurements.

This study developed and validated a novel nomogram that integrates endometrial morphology, hemodynamics, and uterine mechanics. The high predictive performance of this model (AUC = 0.828) stems from its holistic evaluation of the implantation microenvironment: ET reflects the “soil”, RI, S/D reflect the “metabolic supply”, and uterine peristalsis indicates “mechanical stability.” By integrating these distinct physiological dimensions, the proposed model offers a reliable, non-invasive tool to refine clinical decision-making and improve pregnancy outcomes in FET cycles.

## Data Availability

The raw data supporting the conclusions of this article will be made available by the authors, without undue reservation.
